# Allopatric divergence of *Stuckenia filiformis* (Potamogetonaceae) on the Qinghai-Tibet Plateau and its comparative phylogeography with *S*. *pectinata* in China

**DOI:** 10.1038/srep20883

**Published:** 2016-02-11

**Authors:** Zhi-Yuan Du, Qing-Feng Wang

**Affiliations:** 1 Key Laboratory of Aquatic Botany and Watershed Ecology, Wuhan Botanical Garden, Chinese Academy of Sciences, Wuhan 430074, China

## Abstract

In the aquatic genus *Stuckenia*, the wide geographic range of *S*. *pectinata* and *S*. *filiformis* make them suited for examination of topographic and climatic effects on plant evolution. Using nuclear ITS sequence and ten chloroplast sequences, we conducted comparative phylogeographical analyses to investigate their distribution regions and hybrid zones in China, and compare their phylogeographical patterns and demographical histories. These two species were allopatric in China. *S*. *filiformis* occurred only on the Qinghai-Tibet Plateau (QTP), whereas *S*. *pectinata* occupied a wide range of habitats. These two species formed hybrid zones on the northeastern edge of QTP. Most of the genetic variance of *S*. *filiformis* was between the southern and eastern groups on the QTP, showing a significant phylogeographic structure. The geographical isolations caused by the Nyenchen Tanglha Mountains and the Tanggula Mountains promoted intraspecific diversification of alpine plants on the QTP. This study revealed the lack of phylogeographic structure in *S*. *pectinata*, due to the continued gene flow among its distribution regions. The ecological niche modeling showed that the distribution ranges of these two herbaceous species did not contract too much during the glacial period.

Phylogeographical patterns in China did not show an expected contraction-expansion pattern, which is in accordance with the geological records showing that no unified ice sheet had developed in China during the Quaternary^1^. Instead, intraspecific divergences, regional expansions, and hybridizations between these intraspecific lineages were common during the Quaternary climatic oscillations in China. Hybridizations and introgressions between closely related species were also frequently detected at the hybrid zones of two diverging species with different preferences of the ecological niches^2^.

China consists of the following five major regions: north China, subtropical (central/east/south) China, southwest China (Yungui Plateau), northwest China and Qinghai–Tibetan Plateau (QTP). In north, subtropical and southwest China, the predominant response of temperate plant species to Quaternary climatic change appeared to be one of range fragmentation, vicariance, and population isolation with limited or no spatial demographic expansion^3^. In north China, some temperate species migrated to the south, while some species had northern refugia at higher latitudes. Species in the arid northwestern China are often sparsely distributed and ancient. The deserts might have promoted the allopatric divergences of species distributed in this region^1^.

Species distributions in the western high mountain areas of China were affected by drastic changes in topography and altitude due to the uplift of the QTP^4^. The QTP is the highest (averaging about 4500 m) and largest (2.5 × 10^6^ km^2^) plateau on earth^5^. It started to uplift after the collision between India and Eurasia about 50 million years ago (Ma). However, geological estimates date the start of extensive uplifts within the mid-Miocene (c. 15-10 Ma)^4^. A large ice-sheet covering the whole QTP has never existed during the Quaternary glacial^6^. There was phylogeographical evidence for both postglacial re-colonization of the QTP from eastern glacial refugia, and glacial *in situ* survival on the central platform^3^.

Few phylogeographical studies in China were conducted on herbaceous aquatic species. Aquatic plants are widespread compared to terrestrial plants. Many aquatic species are globally distributed, such as *Ceratophyllum demersum* and *Stukenia pectinata*. Most of the widespread aquatic species excel in asexual reproduction with a variety of clonal propagules. They are also capable of long distance dispersal by seeds and specialized vegetative structures^7^. The aquatic genus *Stuckenia* (Potamogetonaceae) is characterized by long stipular sheathes, tubular leaves with air channels bordering the midrib, flexuous peduncles, and subterranean tubers^8^. The only cosmopolitan species in this genus is *S. pectinata* (L.) Börner, while *S*. *filiformis* (Persoon) Börner is mostly distributed in America and Europe, and extends to Western, Northern and Central Asia^9^.

*Stuckenia* species show a wide range of morphological variation. The most reliable characters which separate these two species are the structure of the leaf sheaths and the size of the fruits^8^. In *S. pectinata* the margins of the sheaths are free and often overlap, and the fruits are longer than 3.3 mm. *S. filiformis*, by contrast, has sheaths with margins which are united to form a tube, and fruits which are usually shorter than 3 mm, and do not exceed 3.2 mm^10^. Their hybrid *S.* × *suecicus* has been described by Hollingsworth *et al.*^8^ and Preston *et al.*^10^. However, because of similar vegetative characters, *Stuckenia* hybrids are extremely difficult to distinguish from their highly variable parental species. Thus molecular techniques have been used to investigate the nature of some putative *Stuckenia* hybrids^8^,^10^.

Phylogeography is concerned with principles and processes governing the geographical distributions of genealogical lineages, especially those within and among closely related species^11^. The wide geographical range of *S*. *pectinata* and *S*. *filiformis* make them suited for examination of topographic and climatic effects on plant evolution and speciation. Herein we report on the comparative phylogeography of *S*. *pectinata* and *S*. *filiformis* in China. Chloroplast (cp) DNA is maternally inherited in Potamogetonaceae by experimental hybrids^12^ and is therefore a good marker for tracing range expansion. Internal transcribed spacer (ITS) of nuclear ribosomal DNA is suitable for DNA barcoding in Potamogetonaceae^13^. So in the present study, nuclear ITS sequence and ten chloroplast sequences were used to examine the phylogeographical pattern of populations of *S*. *pectinata* and *S*. *filiformis* across their distribution ranges in China.

The specimen records showed that these two species were allopatric in China^14^. *S*. *pectinata* were mainly distributed in the temperate regions and the Yungui Plateau (YGP), whereas its specimens were rare in subtropical China and the QTP. In contrast, *S. filiformis* were mainly distributed on the QTP. However, its specimens were scarcely found on the high altitudinal and arid northwestern QTP, and were mainly restricted to the middle altitudinal and moist southern and eastern QTP. Our specific objectives were to use DNA barcoding, ecological niche modeling and phylogeographical analysis to (1) investigate the distribution regions of these two species in China and find whether they have sympatric regions and hybrid zones, and (2) compare their phylogeographical patterns and demographical histories as two closely related aquatic species.

## Results

### Nuclear genotype variation and distribution

The nuclear genotypes of each sampling population were recorded in Supplementary Table S1 and S2. The length of the alignment of ITS sequences of *S*. *filiformis* and *S*. *pectinata* were 682 bp and 673 bp respectively. A total of 31 base substitutions and two length variants were found between these two species (Supplementary Table S3). Only one ITS genotype was found in *S*. *filiformis*, while nine polymorphic sites were detected in *S*. *pectinata* defining three homozygous genotypes (AA, BB and CC). Three heterozygous genotypes (AB, AC, BC) with additive pattern of ITS sequences were also generated in *S*. *pectinata* by cloning their ITS sequences. Each population of *S*. *filiformis* and *S*. *pectinata* only had one ITS genotype. All hybrid individuals of *S.* × *suecicus* (*S. filiformis* × *S. pectinata*) possessed an additive pattern of ITS sequences. When sequences were cloned from them, these hybrid individuals yielded two types of ITS sequences, which were identical to either *S*. *filiformis* or *S*. *pectinata*.

**Figure 1:**
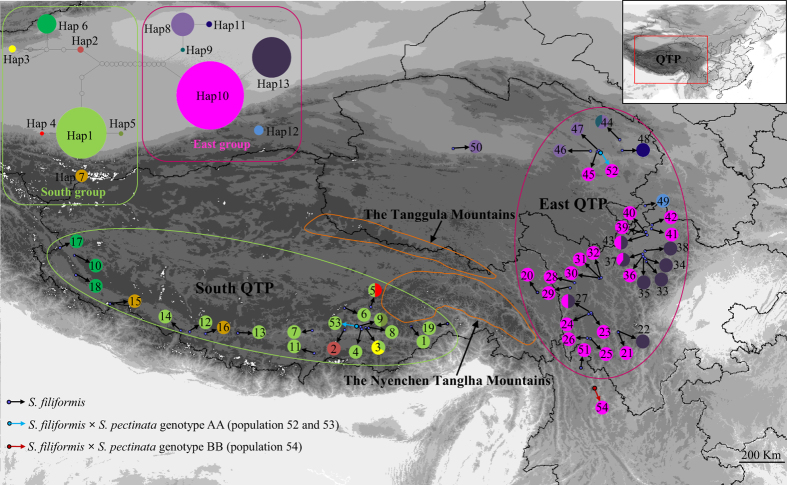
The geographical distribution of *S. filiformis* populations. The map is created by DIVA-GIS. Right top is the map of China, indicating the Qinghai-Tibetan Plateau. Sampling locations are marked with population codes. Populations indicated by red and blue arrows are hybrids between *S. filiformis* and *S. pectinata*. Pie charts show the proportions of haplotypes within each population. Solid lines encircle SAMOVA-identified groups: green, ‘South group’; and red, ‘East group’. Left top is the network of 13 haplotypes of *S. filiformis*. The pie size is proportional to the haplotype frequency. Each dot between haplotypes indicates a mutational step.

**Figure 2:**
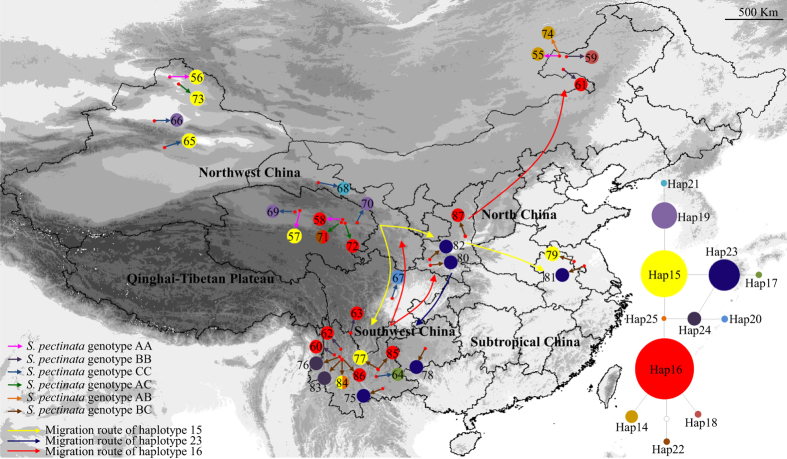
The geographical distribution of *S. pectinata* populations. The map is created by DIVA-GIS. Sampling locations are marked with population codes. Pie charts show the proportions of haplotypes within each population. The nuclear genotypes of *S. pectinata* are indicated by short arrows of different colors. Long arrows indicate the migration routes of widespread haplotypes. Right bottom is the network of 12 haplotypes of *S. pectinata*. The pie size is proportional to the haplotype frequency. Each dot between haplotypes indicates a mutational step.

The geographic distributions of nuclear genotypes of *S*. *filiformis* and *S*. *pectinata* were shown in Figs 1 and 2. Their altitudinal distributions were shown in Supplementary Figs S1 and S2. In China, *S*. *filiformis* did not occur outside of the QTP. In accordance with the specimen records, *S. filiformis* had a discontinuous distribution on the QTP. It scarcely distributed on the high altitudinal and arid northwestern QTP. This species mainly occurred at intermediate altitudes ranging from 3000–4800 m on the southern and eastern plateau. *S*. *pectinata* occupied a wide range of habitats in northwest China, north China and southwest China (Fig. 2). On the QTP, *S*. *pectinata* occurred only at lower altitudinal northeastern edge of the plateau. This species did not occur in subtropical China. Both the homozygous genotypes (AA, BB and CC) and heterozygous genotypes (AB, AC, BC) of *S*. *pectinata* were common in China (Fig. 2). The hybrid *S.* × *suecicus* (*S. filiformis* × *S. pectinata*) occurred in the northwestern YGP and the northeastern and southern QTP (Fig. 1).

### Chloroplast haplotype variation and distribution

Both species were sequenced for the ten cpDNA regions. The total alignment length of the cpDNA sequences was 6819 bp. These two species had 128 polymorphic sites in the combined sequences, including 104 substitutions and 24 indels. For each cpDNA region, the two species had many interspecies variable sites. However, not every sequence was polymorphic within each species. Five cpDNA regions, *atp*F-*atp*H, *rpl*20-*rps*12, *trn*D-*trn*T, *trn*S-*trn*G and *trn*L-*trn*F, showed polymorphisms in *S*. *filiformis*, whereas other five cpDNA regions, *trn*L-*rpl*32, *trn*Q-*rpS*16, *ndh*Ax1-*ndh*Ax2, *rpo*B-*trn*C and *psb*M-*trn*D, showed polymorphisms in *S*. *pectinata*. Twenty-nine polymorphic sites were found in *S*. *filiformis*, while nine substitutions and two indels were found in *S*. *pectinata* (Supplementary Table S4 & S5).

Thirteen and 12 haplotypes were revealed in *S*. *filiformis* and *S*. *pectinata* respectively. Haplotypes in each population of *S*. *filiformis* were presented in Supplementary Table S1. Among 51 populations of *S*. *filiformis*, 44 were fixed by a single haplotype, while the remaining seven were polymorphic. Haplotypes in the 33 investigated populations of *S*. *pectinata* were listed in Supplementary Table S2. Only two populations in the 33 populations of *S*. *pectinata* were polymorphic, while the remaining 31 populations were fixed for a single haplotype. The haplotype diversity (*H*_*d*_) of *S*. *filiformis* and *S*. *pectinata* were estimated to be 0.797 and 0.776 respectively.

The haplotype distributions of *S*. *filiformis* were structured into two geographical regions: the southern and eastern QTP (Fig. 1). Nineteen populations from the southern plateau harboured seven haplotypes (Hap1–7). Thirty populations from the eastern plateau harboured six haplotypes (Hap8-13). The geographical distributions of *S*. *pectinata* haplotypes were shown in Fig. 2. Hap15 and Hap16 were the most widespread haplotypes, occurring in northwest, north and southwest China. Hap23 also had a wide spread distribution in north China and southwest China. Some unique haplotypes were found in northwest China (Hap19, 21 & 22), north China (Hap14 & 18) and southwest China (Hap17, 20, 24 & 25).

### Ecological niche modeling

**Figure 3:**
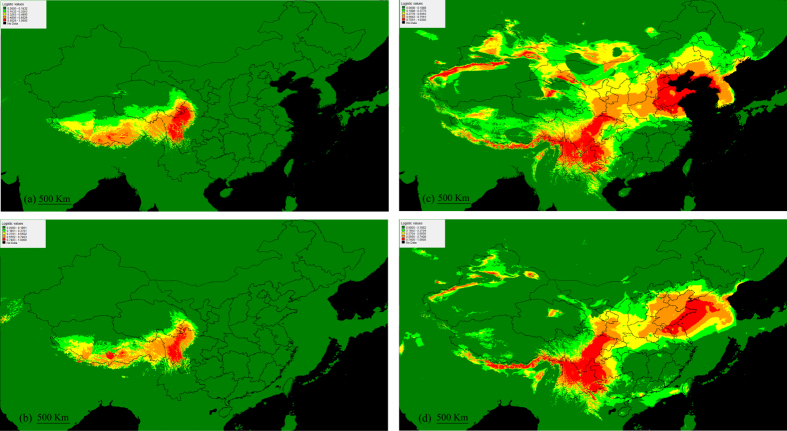
Ecological niche modeling for *S*. *filiformis* and *S*. *pectinata* in China. The maps are created by MAXENT and DIVA-GIS. The strength of prediction is indicated according to the key shown. Red and orange areas show strong predictions. (**a**) Regions in China considered suitable for *S*. *filiformis* under current climate conditions. (**b**) The paleodistribution model for *S*. *filiformis* in the LGM (22,000 years ago). (**c**) Regions in China considered suitable for *S*. *pectinata* under current climate conditions. (**d**) The paleodistribution model for *S*. *pectinata* in the LGM (22,000 years ago).

Based on binomial tests of omission, the models produced predictions that were significantly better than random (*P* *<* 0.001). The ecological niche models under current climate conditions differed greatly between species (Fig. 3). For both species, the palaeodistribution models showed that the habitats predicted as suitable during the LGM were a little smaller than the current distributions (Fig. 3).

### Haplotype network and phylogeny

**Figure 4:**
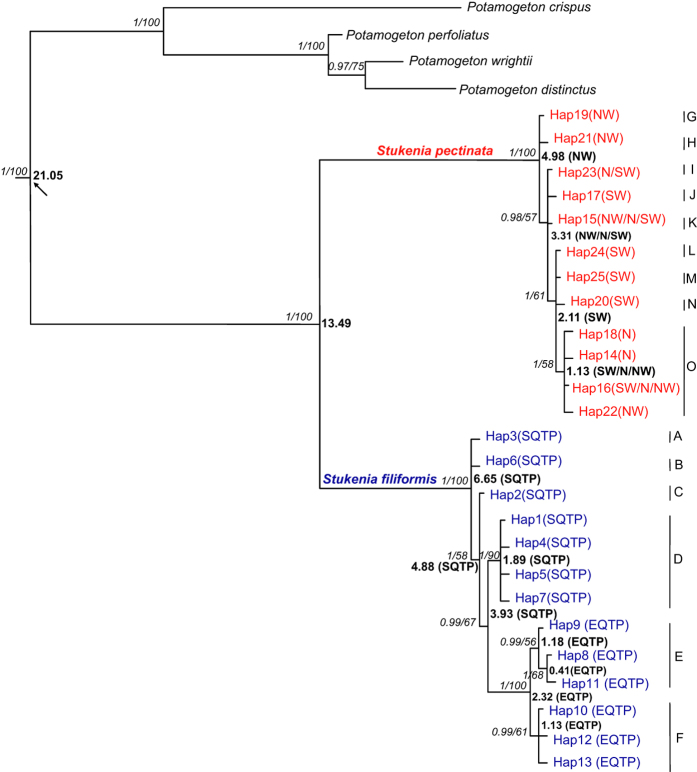
Phylogenetic relationships of haplotypes. Support values (Bayesian posterior probability /maximum likelihood bootstrap) are next to the nodes. Arrow denotes node where fossil calibration was applied. The divergence times and ancestral areas are shown at nodes. The time scale is in Ma. The distributions were categorized into the following areas: northwest China (NW), north China (N), southwest China (SW), south QTP (SQTP) and east QTP (EQTP).

The genealogical relationships among the haplotypes were revealed in the parsimony networks (Figs 1 and 2). Bayesian and maximum likelihood (ML) analyses produced highly consistent tree topologies (Fig. 4). The support values of both Bayesian posterior probability and maximum likelihood bootstrap were annotated in the phylogenetic tree (Fig. 4). Phylogenetic analyses showed that both species were monophyly (Fig. 4). There were six clades in *S*. *filiformis*. The first four clades (A, B, C & D) comprised seven haplotypes (Hap1-7) located at the southern plateau, whereas the rest two clades (E & F) comprised six haplotypes (Hap8-13) situated in the eastern plateau. In the haplotype network, these two geographical lineages were separated by 14 steps (Fig. 1). Nine clades were identified in *S*. *pectinata* (Fig. 4). Clade G and H included two haplotypes (Hap19 & 21) distributed in northwest China (including part of the northeastern QTP). The rest clades consisted of ten haplotypes distributed in northwest, north and southwest China.

### Divergence time estimation and ancestral area reconstruction

The estimated divergence time between *S*. *filiformis* and *S*. *pectinata* was 13.49 Ma (Fig. 4). The two geographical lineages of *S*. *filiformis* diverged from 3.93 Ma. The reconstructed ancestral areas were shown in Fig. 4. RASP analyses suggested that *S*. *filiformis* migrate from the southern plateau (clades A, B, C & D) to the eastern plateau (clades E & F). The reconstruction results showed that *S*. *pectinata* migrated from northwest China (clades G & H) to north and southwest China (clades I, J & K). It diversified in southwest China (clades L, M & N), and then dispersed from southwest China to north and northwest China (clade O). The migration routes of the widespread haplotypes (Hap15, 23 &16) were annotated on the distribution map of *S*. *pectinata* (Fig. 2).

### Population structure and genetic variation

Results of the AMOVA analyses were presented in Supplementary Table S6. About 96.37% of the total variation of *S*. *filiformis* was partitioned among populations, whereas just 3.63% was within populations. When populations were grouped according to geographical region, AMOVA revealed that approximately 89% of the total genetic variation was assigned between the southern and eastern QTP, while only 9.11% of variation occurred among populations within these regions. The *F*_*ST*_ value of *S*. *filiformis* was 0.981, and the gene flow (*N*_*m*_) was 0.01 individual per generation. The AMOVA results indicated that 75.54% of the total genetic variation of *S*. *pectinata* occurred among populations and 24.46% within populations. When considering groupings based on geographical range (northwest, north and southwest China), we detected little variation among groups (7.46%). The *F*_*ST*_ and *N*_*m*_ values of *S*. *pectinata* were 0.755 and 0.16 respectively.

### Phylogeographical structure

Permutation test of *S*. *filiformis* showed that *N*_*ST*_ (0.982) was significantly higher than *G*_*ST*_ (0.886; *P* *<* 0.05), indicating significant phylogeographical structure across the species’ entire distributional range. Spatial genetic analysis of *S*. *filiformis* using SAMOVA revealed the presence of two groups of populations with an *F*_*CT*_ value of 0.882. This grouping is mostly consistent with genealogical relationships of haplotypes (Fig. 1). Populations 1–20 comprised the southern plateau group, while the remaining populations 21–51 belonged to the eastern plateau group (Fig. 1). However, within these two groups, *N*_*ST*_ was not significantly higher than *G*_*ST*_ (*P* *<* 0.05). This result implies that if any phylogeographical structure was present within either group of *S*. *filiformis*, it was very weak. The permutation test of *S*. *pectinata* showed that *N*_*ST*_ (0.977) was higher than *G*_*ST*_ (0.954), but not significantly (*P* > 0.05), implying a lack of correlation between haplotypes and geographical distribution^15^. The SAMOVA analysis failed to uncover any reliable groups from populations of *S*. *pectinata*.

### Demographic history

**Figure 5:**
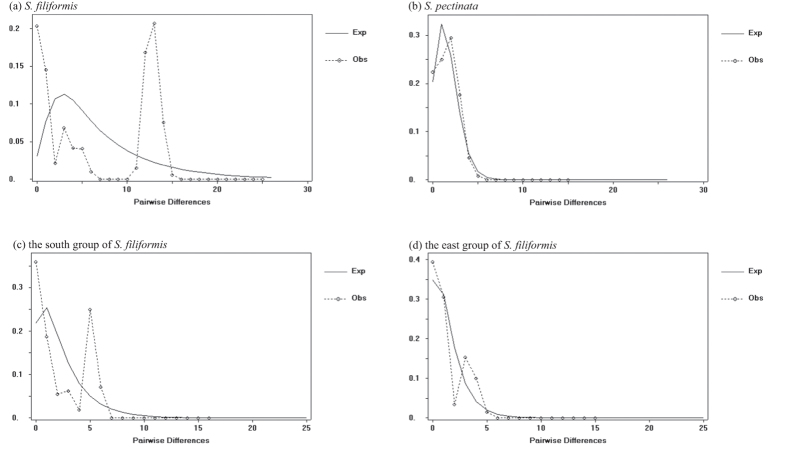
Mismatch distributions of the number of pairwise nucleotide differences of *S*. *filiformis* (**a**), *S*. *pectinata* (**b**) and the south and east groups of *S*. *filiformis* (**c**,**d**). The dashed line represents observed values (Obs) whereas the solid line shows expected values (Exp) under the population growth-decline model. The Y axis is frequency.

Results of the Fu’s *Fs* test and Tajima’s *D* test were presented in Supplementary Table S7. Positive Fu’s *Fs* value (6.517; *P* *<* 0.05) and non-significant Tajima’s *D* value (2.028; *P* > 0.05) supported that *S*. *filiformis* did not experience rapid range expansion. Its 95% confidence intervals of growth rates included zero (Supplementary Table S7), which indicated that there was little or no growth in *S*. *filiformis*. The observed mismatch distributions of *S*. *filiformis* also differed from sudden range expansion (Fig. 5). For both geographical groups of *S*. *filiformis*, the deviation in mismatch distribution (Fig. 5) and non-significant Tajima’s *D* and Fu’s *Fs* values (Supplementary Table S7) indicated that neither group had experienced sudden range expansion.

The observed mismatch distributions of *S*. *pectinata* fitted the population growth-decline model (Fig. 5). However, the tests of neutrality for *S*. *pectinata* showed that Fu’s *Fs* value was slightly negative but not significant (−0.579; *P* > 0.1) and Tajima’s *D* value was non-significant (0.2452; *P* > 0.1). In addition, its 95% confidence intervals of growth rates included zero (Supplementary Table S7). These results indicated that there was some growth in *S*. *pectinata*, even though it was not an sudden range expansion. In the three distribution regions of *S*. *pectinata*, the results of mismatch distribution analyses (Supplementary Fig. S3) and neutral tests (Supplementary Table S7) provided no evidence of rapid range expansion in these regions.

## Discussion

*S*. *pectinatus* is the most widespread species of *Stuckenia* and occurs in all continents of the world. *S*. *filiformis*, in contrast, is primarily a circumboreal species of more northerly latitudes^8^. In this study, DNA barcoding using ITS sequences revealed that only one nuclear genotype was found in *S*. *filiformis*, whereas three homozygous genotypes and three heterozygous genotypes were detected in *S*. *pectinata*. The geographical distributions of *S*. *filiformis* in China showed that this cold-tolerant species had a discontinuous distribution on the QTP (Fig. 1). The ecological niche model (Fig. 3) was consistent with the specimen records^14^. *S. filiformis* scarcely distributed on the high altitudinal and arid northwestern QTP, and mainly occurred at intermediate altitudes ranging from 3000–4800 m on the moist southern and eastern plateau. The Nyenchen Tanglha Mountains and the Tanggula Mountains located between the southern and eastern populations of *S*. *filiformis* (Fig. 1).

In contrast, *S*. *pectinata* occupied a wide range of habitats in temperate regions, whereas in subtropical region it is only found on the Yungui plateau (averaging about 2000 m) and the adjacent hills (Fig. 2). In the QTP, *S*. *pectinata* occurred only at altitudes ranging from 2500–3200 m on the northeastern edge of the plateau. The ecological niche model (Fig. 3) and the specimen records showed the same distributions.

These two species could hybridize in sympatric regions, even though their hybrids are sterile and propagate by asexual reproduction^8^,^10^. Hybridization between these two species was detected at the hybrid zone of Daotang River (population 52, 3143 m asl) on northeastern QTP, the only region where both species were found. The hybrid *S*. × *suecicus* were also found in other areas with only one parental species. In these regions, the disappeared parental species may once disperse to there and leave some relic populations.

In the southern valley of QTP (population 53, 3642 m asl), the hybrid *S*. × *suecicus* co-occurred only with *S*. *filiformis*. The ecological niche model (Fig. 3) predicted that *S*. *pectinata* could live along the southernmost edge of QTP (the Yarlung Tsangpo Grand Canyon in southeastern QTP and the south slope of the Himalayas). These results suggested that some populations of *S*. *pectinata* might disperse into the southern valley of QTP along the Yarlung Tsangpo River and hybridize with *S*. *filiformis* there. The cpDNA haplotype of this *S*. × *suecicus* population (Hap 1) is identical to the southern populations of *S*. *filiformis*. Because no *S*. *filiformis* population on the northeastern QTP has the same haplotype, this population of *S.* × *suecicus* could not be the descendents migrated from the hybrid zone on the northeastern QTP.

In the northwestern YGP (population 54, 2435 m asl), *S*. × *suecicus* lived with *S*. *pectinata*, while *S*. *filiformis* was missing. The postglacial retreat of *S*. *filiformis* from the northwest YGP was predicted by the ecological niche model (Fig. 3). The cpDNA haplotype (Hap 10) of this *S.* × *suecicus* population was identical to the eastern populations of *S*. *filiformis*, and the genotype of its paternal parent (genotype BB) was identical to the northwest YGP populations of *S*. *pectinata*. Because no *S*. *pectinata* population on the northeastern QTP is genotype BB, this population of *S.* × *suecicus* could not be the emigrants from the hybrid zone on the northeastern QTP.

The three homozygous genotypes of *S. pectinata* were sympatric, which led to the extensive hybridization and introgression among these genotypes (Fig. 2). If *S. filiformis* and *S. pectinata* are sympatric, they would hybridize frequently. Allopatric distribution is one of the mechanisms of geographical isolation between species that have incomplete reproductive isolation. It can reduce their hybridization frequency and keep them as two independent species.

Both species had high total genetic diversity at the species level (*h*_*T*_ = 0.806 for *S*. *filiformis* and 0.876 for *S*. *pectinata*), whereas the average haplotype diversity within population was low (*h*_*S*_ = 0.092 for *S*. *filiformis* and 0.04 for *S*. *pectinata*). This is because most of the *S*. *filiformis* and *S*. *pectinata* populations only contain a single haplotype, which is common in clonal aquatic plants^7^,^8^,^16^. However, the levels of genetic differentiation were different between these two species. Approximately 89% of the total genetic variation in *S*. *filiformis* was assigned between the east and south groups. Because of the marked geographical structure, a very high estimate of interpopulation differentiation was recorded (*F*_*ST*_ = 0.981) in *S*. *filiformis*. In contrast, the genetic differentiation was lower (*F*_*ST*_ = 0.755) in *S*. *pectinata*, due to the lack of differentiation among its distribution regions (northwest, north and southwest China). Only 7.46% of the cpDNA variation in *S*. *pectinata* was distributed among the three distribution regions. Comparing to the extremely low level of gene flow in *S*. *filiformis* (*N*_*m*_ = 0.01), *S*. *pectinata* showed a higher level of gene flow (*N*_*m*_ = 0.16).

PERMUT analysis suggested a distinct phylogeographical structure in *S*. *filiformis* (*G*_*ST*_ *<* *N*_*ST*_; *P* *<* 0.05). SAMOVA identified that most of the genetic variance was between the south and east groups (Fig. 1). This was largely due to the major differences in haplotype composition between populations on the eastern and southern plateau. Haplotype network also showed that *S*. *filiformis* comprises two major lineages. The deep lineages within *S*. *filiformis* were dated to occur during the Pliocene (3.93 Ma) far before the LGM. Populations from the southern plateau were found to harbour ancestral haplotypes. Divergence time estimation and ancestral area reconstruction suggested that *S*. *filiformis* migrate from the southern plateau to the eastern plateau during the Pliocene (Fig. 4). After this migration, topographic changes on the QTP may have caused the intraspecific divergences through allopatric differentiation. The rising of the Nyenchen Tanglha Mountains and the Tanggula Mountains in the southeastern and central plateau may have blocked the gene flows between these two groups, and triggered the genetic divergence between them.

The Nyenchen Tanglha Mountains is a 700 km long mountain range located in the southeastern QTP^17^. The rugged and heavily glaciated range counts more than 240 peaks over 6000 m. It contains 7080 glaciers covering an area of 10700 km^2^. There are 32 glaciers that are over 10 km long^17^. In central QTP, the Tanggula Mountains locate at the north of Nyenchen Tanglha Mountains. The elevations of its main ridge are above 5000 m, and Peak Geladandong (6621 m) is the tallest in the range. The extremely low temperature on these high mountains and development of glaciers have blocked gene flow between geographically isolated east and south groups of *S*. *filiformis* and promoted its deep intraspecific divergence. Because the diverged lineages caused by this geographical isolation have not contact again, intraspecific introgressions between them were not found.

This allopatric divergence was also recovered for another alpine shrub species^18^. Phylogeographical analysis of *Hippophae tibetana* showed that it comprised two lineages, distributed in the eastern and southern QTP. The estimated timescale for the deep divergences within this species was 4 Ma if the slow evolutionary rate was adopted. In this study, the two geographical lineages of *S*. *filiformis* also diverged from 3.93 Ma. These two studies provided evidence that during the Pliocene geographical isolations caused by high mountains, cold climate and glaciers promoted intraspecific diversification of alpine plants on the QTP.

*S*. *pectinata* distributed in northwest China, north China and southwest China. The three homozygous nuclear genotypes regained contact, which led to frequent hybridizations and the share of haplotypes between them. Genotype AA and BB shared Hap16, while genotype AA and CC shared Hap 15. These two haplotypes were the most widespread haplotypes, occurring in northwest, north and southwest China. The heterozygous genotype BC had three unique haplotypes (Hap23, 24 & 25), and Hap23 was widely spread in north China and southwest China.

PERMUT analysis recorded no significant phylogeographical structure in *S. pectinata* (*G*_*ST*_ *<* *N*_*ST*_, *P* > 0.05), which suggested that considerable gene flow occur among the three distribution regions. All the haplotypes of *S. pectinata* occurred before the LGM, but only the populations from northwest China were found to harbour ancestral haplotypes (Hap 19 & 21). Molecular dating and RASP analyses suggested that their descendent haplotype (Hap15) migrate from northwest China to north China and southwest China during the Pliocene (Fig. 4). After this migration, *S. pectinata* diversified in the YGP (Hap 20, 24 & 25) during the Pleistocene, and then one new haplotype (Hap 16) migrated backwards from southwest China to north China and northwest China. Followed by this migration, some new haplotypes were formed in northwest China (Hap 22) and north China (Hap 14 & 18). The lack of phylogeographical signal was mostly due to the facts that *S*. *pectinata* exhibited a continuous distribution of dominant haplotypes (Hap15, 16 & 23) among its distribution regions, and the gene flows among these regions continued from the Pliocene to the Pleistocene. The lack of geographical isolation among these regions and the ability of long distance dispersal triggered these gene flows among populations of *S*. *pectinata*.

Because of arid conditions and complex topography, QTP glaciers were less prominent than other regions in the Northern Hemisphere^6^. Potential habitats for cold-tolerant species could be found in the plateau during the ice ages^19^. The palaeodistribution model predicted that *S*. *filiformis* maintained in the southern and eastern QTP during the LGM (Fig. 3). At the end of the LGM, we failed to detect the large-scale range expansion of *S*. *filiformis*. Mismatch distribution of pairwise difference among haplotypes and Fu’s *Fs* and Tajima’ *D* statistic suggested that the whole species and both of the two groups of *S*. *filiformis* underwent no interglacial or postglacial rapid range expansion. Some other alpine species experienced the same demographic histories on the QTP^18^,^20^,^21^. The Quaternary glaciations reinforced the geographical isolation by the high mountains between the south and east groups of *S*. *filiformis*. After the LGM, they were still isolated by these high mountains. Until now, there is no sign that these two groups have gene flows between them.

The geological records showed that no unified ice sheet had developed in China during the Quaternary Period, so the phylogeographical patterns in China did not show an expansion-contraction pattern at large scales^1^. The palaeodistribution model predicted that during the LGM *S. pectinata* persisted in high latitudinal northwest China and north China, and low latitudinal southwest China (Fig. 3). But its distribution range in the northwest and north China has slightly contracted. This is in accordance with the results of the mismatch distribution analysis and the neutrality tests, indicating that there was some growth in *S*. *pectinata*, even though the whole species and the three distribution regions of *S*. *pectinata* experienced no rapid range expansion. The distribution pattern of *S. pectinata* differs from large-scale range expansion, in which the haplotype diversity gradually decrease with increasing distance to the southern refugia^22^. Instead, our result is consistent with demographic history of another widespread herb *Clintonia udensis* in East Asia, which showed no signature of rapid range expansion^23^.

This comparative study of *Stuckenia* species is one of a few studies that have been conducted on widespread herbaceous species in China. It provided evidence that geographical isolations caused by the Nyenchen Tanglha Mountains and the Tanggula Mountains promoted intraspecific diversification of alpine plants on the QTP. It also highlighted the importance of northwest China and southwest China in the evolutionary history of widespread temperate species in China. The populations from northwest China could harbor ancestral haplotypes, and migrate to north China and southwest China (after the uplift of YGP), whereas new haplotypes could diversify in the YGP, and migrate backwards to north China and northwest China. Because the northern part of China and the whole QTP were not covered by large ice sheets during the Quaternary glaciations, some plant species could survive in the north region of China (temperate species) and the QTP (cold-tolerant species) during the glacial period. This comparative study would provide insights into the evolutionary history of plants with similar life histories and distribution patterns in China.

## Methods

### Population sampling

A total of 87 populations of *Stuckenia* species were collected throughout the entire range of this genus in China. For each population, 5–10 randomly selected individuals were sampled at intervals of at least 10 m apart to avoid collecting clones. Fresh leaves were immediately dried in silica gel. Voucher specimens were deposited at Wuhan Botanical Garden, Chinese Academy of Sciences. The latitude, longitude, and altitude of each sampling site were recorded in Supplementary Table S1 and S2. *Stuckenia* was recently elevated from a subgenus in *Potamogeton* to the rank of genus^14^. Because we need to use the known fossil ages of *Potamogeton* for divergence time estimations, we selected four *Potamogeton* species (*P*. *crispus*, *P*. *distinctus*, *P*. *wrightii*, and *P*. *perfoliatus*) as outgroups for phylogenetic analyses.

### DNA extraction, PCR amplification and sequencing

Total genomic DNA was extracted from silica-gel-dried leaf tissue using a modified CTAB method^24^. Both ITS1 and ITS2 regions of ITS sequence^25^ and ten cpDNA regions (*trn*L-*trn*F^26^; *trn*D-*trn*T^27^; *rpl*20-*rps*12, *trn*S-*trn*G^28^; *rpo*B-*trn*C, *psb*M-*trn*D^29^; *trn*L-*rpl*32, *trn*Q-*rpS*16, *ndh*Ax1-*ndh*Ax2^30^; *atp*F-*atp*H^31^) were amplified and sequenced for all the samples of both species using universal primers. The procedures for PCR amplification and sequencing were following Du *et al.*^13^. If superimposed nucleotides (additive patterns) were found in the chromatograms of ITS sequences, the purified PCR products were cloned into the pUC19 vector (TaKaRa). For each of them, five positive clones were sequenced.

### Nuclear genotype and chloroplast haplotype variation and distribution

We used D_NA_SP v.5.0 to extract nuclear genotypes and chloroplast haplotypes^32^. The sequences of nuclear genotypes and cpDNA haplotypes were deposited in GenBank under accession numbers KP676469 - KP676566. Haplotype diversity (*H*_*d*_) were calculated using D_NA_SP. The geographical and attitudinal distributions of genotypes and haplotypes were plotted on a relief map of China using DIVA-GIS^33^.

### Ecological niche modeling

The global climate database from WorldClim^34^ was used for ecological niche modeling under current climate conditions using MAXENT v.3.3.3^35^. Climate estimates for the last glacial maximum (LGM) was provided by the Palaeoclimate Modelling Intercomparison Project Phase II (PMIP2). For each species, a distribution model was generated using 19 bioclimatic parameters for the current climate and the collection localities. The model was then applied to estimate the geographical distribution for each species during the LGM. Binomial tests of omission were conducted by randomly selecting 25% of the occurrence localities as test data and using 10,000 randomly chosen pixels from the study region as random instances^35^.

### Haplotype network and phylogeny

Genealogical relationships among haplotypes were inferred by TCS v.1.21^36^. Phylogenetic relationships of the two species were estimated by Mrbayes v.3.0^37^. Bayesian analyses were performed over 1.0 × 10^8^ generations under the GTR model with gamma, as inferred from MrModeltest v.2.0^38^. After discarding the first 25% trees as burn in, the remaining trees were used to estimate posterior probability. In addition, phylogenetic relationships were also assessed by maximum likelihood (ML) methods using PhyML v.3.1^39^. The ML analysis was performed using the GTR model with gamma. The robustness of ML tree was tested by 100 bootstrap replicates.

### Divergence time estimation and ancestral area reconstruction

BEAST v.1.5.4^40^was used to estimate the divergence times. The partition homogenetity test revealed no character incongruence among all the ten cpDNA intergenic regions, so we used the combined sequences for analysis. The hypothesis of equal substitution rates among species was tested using log-likelihood ratio test in PAUP^41^, which led to the rejection of the constant molecular clock hypothesis (P *<* 0.05). Thus, an uncorrelated lognormal model was used to describe the relaxed clock. The root of the tree, which is the split between *Potamogeton* and *Stuckenia*, was constrained between 23.03–20.44 Ma based on the known fossils of *Potamogeton*^42^,^43^. Markov Chain Monte Carlo (MCMC) analyses was run for 1.0 × 10^8^ generations under the GTR nucleotide substitution model with gamma. We checked convergence of the stationary distribution using Tracer v.1.5.1^40^, and the effective sample size for each parameter was found to exceed 200. After discarding the first 25% trees as burn in, the samples were summarized in the maximum clade credibility tree using TreeAnnotator v.1.4.8^40^.

We employed the Statistical Dispersal-Vicariance Analysis (S-DIVA) in RASP v.3.0 (Reconstruct Ancestral State in Phylogenies)^44^ to infer the biogeographical history. The geographical distributions of haplotypes were annotated on the phylogenetic tree. We categorized the distributions into the following areas: northwest China (NW), north China (N), southwest China (SW), south QTP (SQTP) and east QTP (EQTP).

### Population structure and genetic variation

Hierarchical analysis of molecular variance (AMOVA) was performed to characterize the population structure and genetic variation using Arlequin v.3.0^45^. To estimate the extent of genetic divergence and gene flow, measures of pairwise genetic differentiation (*F*_*ST*_) and gene flow (*N*_*m*_) among geographical regions were calculated. The Kimura two-parameter model was chosen, and significance of the variance components was tested with 1000 random permutations.

### Phylogeographical structure

Two parameters of differentiation, *G*_*ST*_ (coefficient of genetic variation over all populations) and *N*_*ST*_(equivalent coefficient taking into account sequence similarities between haplotypes) were estimated using the program PERMUT^15^. *G*_*ST*_ and *N*_*ST*_ were compared using a permutation test with 1000 permutations. A higher *N*_*ST*_ than *G*_*ST*_ indicates the presence of phylogeographical structure with closely related haplotypes being found more often in the same area than less closely related haplotypes^15^. Then, spatial genetic structures of haplotypes were analysed by spatial analysis of molecular variance using SAMOVA v.1.0^46^ to define groups of populations that are geographically homogenous and maximally differentiated from each other. The configuration with the largest genetic differentiation (*F*_*CT*_) was retained as the best grouping of populations.

### Demographic history

To detect historical population dynamics, mismatch distribution was estimated. The observed number of differences between pairs of haplotypes was compared to the theoretical distribution using the population growth-decline model with D_NA_SP^32^. The demographic expansion was then tested by D_NA_SP using Fu’s *Fs* test^47^ and Tajima’s *D* test^48^. Significantly negative *Fs* values indicate recent population expansion following a severe reduction in population size^47^, and significant *D* values generally suggest rapid demographic expansions^49^. We also used LAMARC v.2.1^50^ to estimate the growth rates of the two *Stuckenia* species. If the estimate of growth rate is positive and big, but the confidence intervals include zero, it’s quite likely that there is in fact little or no growth^50^. If the intervals exclude zero, that finding is generally reliable^50^. For the two population groups within *S*. *filiformis* (East and South) and the three distribution regions of *S*. *pectinata* (Northwest, North and Southwest), we also performed the mismatch distribution analyses, neutrality tests and coalescent simulations.

## Additional Information

**How to cite this article**: Du, Z.-Y. and Wang, Q.-F. Allopatric divergence of *Stuckenia filiformis* (Potamogetonaceae) on the Qinghai-Tibet Plateau and its comparative phylogeography with *S. pectinata* in China. *Sci. Rep.* **6**, 20883; doi: 10.1038/srep20883 (2016).

## Supplementary Material

Supplementary Information
