# Supplementary material for: Allopatric divergence of Stuckenia filiformis (Potamogetonaceae) on the Qinghai-Tibet Plateau and its comparative phylogeography with S. pectinata in China
Source: Sci Rep. 2016 Feb 11;6:20883. doi: 10.1038/srep20883 (PMC4750007; doi:10.1038/srep20883)
Supplement: Supplementary Information [file srep20883-s1.pdf]

Allopatric divergence of *Stuckenia filiformis* (Potamogetonaceae) on the Qinghai-Tibet Plateau and its comparative phylogeography with *S. pectinata* in China

Zhi-Yuan Du and Qing-Feng Wang

Supplementary Information

Fig. S1 Sampling populations of *S. filiformis* on the altitudinal map of China. The map is created by DIVA-GIS. Sampling locations are marked with violet dots. The range of altitude is indicated according to the key shown.

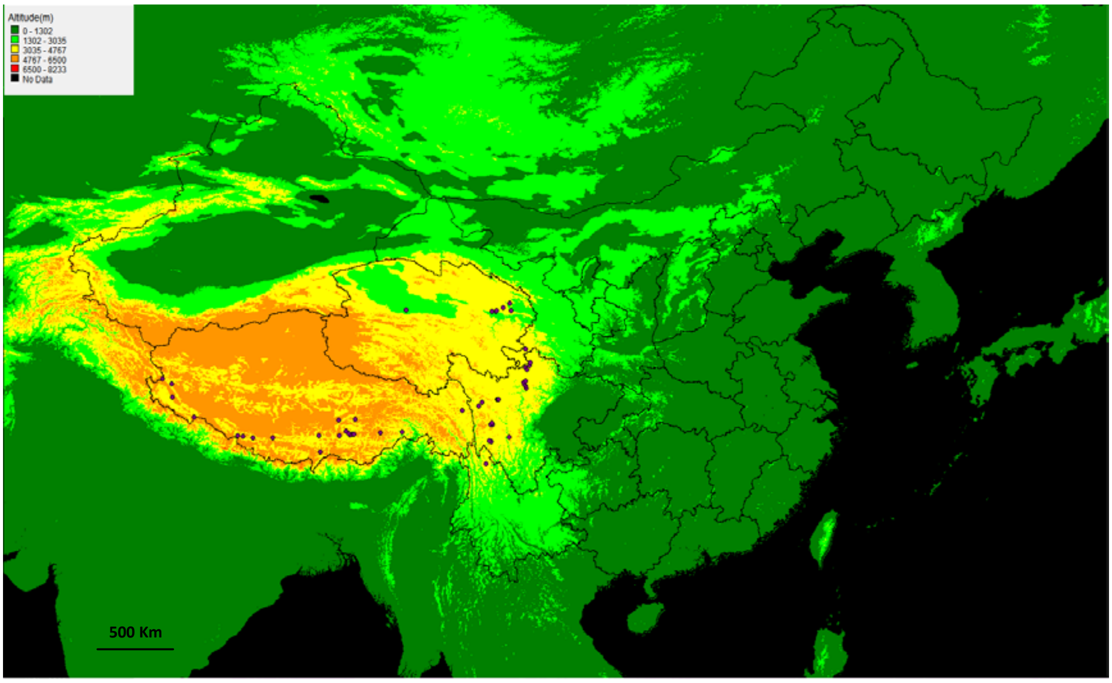

Fig. S2 Sampling populations of *S. pectinata* on the altitudinal map of China. The map is created by DIVA-GIS. Sampling locations are marked with red dots. The range of altitude is indicated according to the key shown.

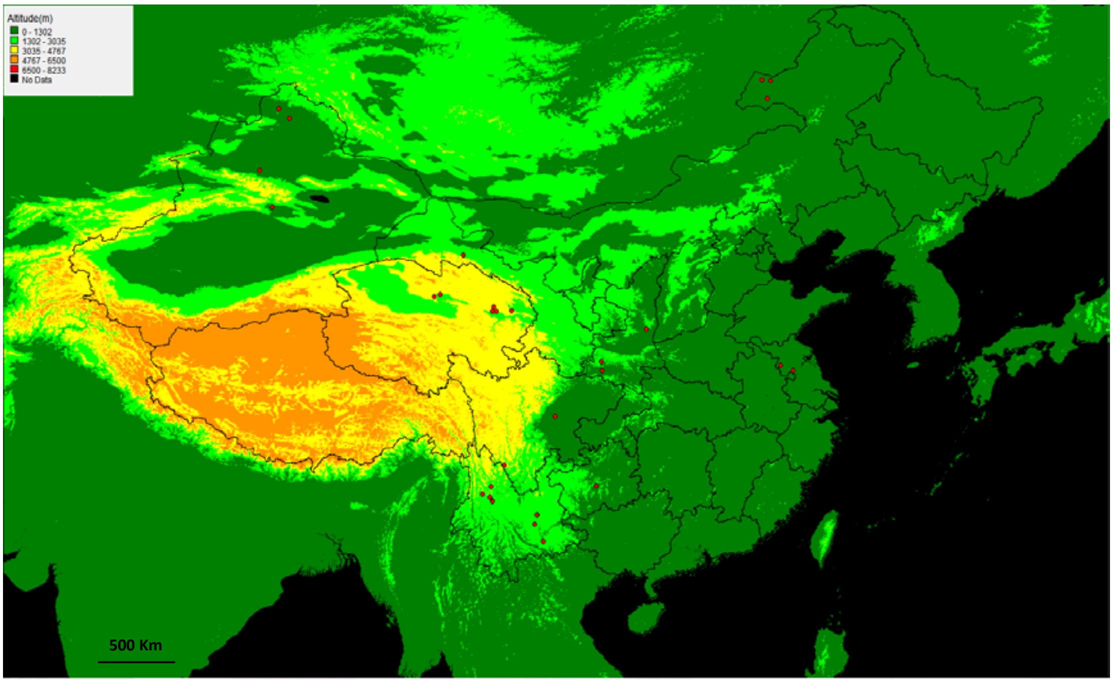

Fig. S3 Mismatch distributions of the number of pairwise nucleotide differences of the northwest, north and southwest regions of *S. pectinata*. The dashed line represents observed values (Obs) whereas the solid line shows expected values (Exp) under the population growth-decline model. The Y axis is frequency.

(a) the northwest region of *S. pectinata*

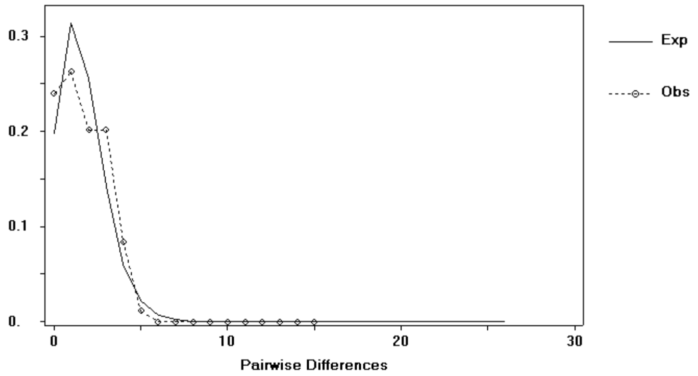

(b) the north region of *S. pectinata*

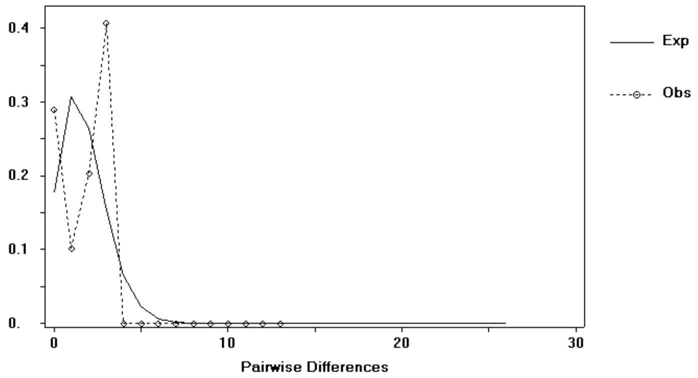

(c) the southwest region of *S. pectinata*.

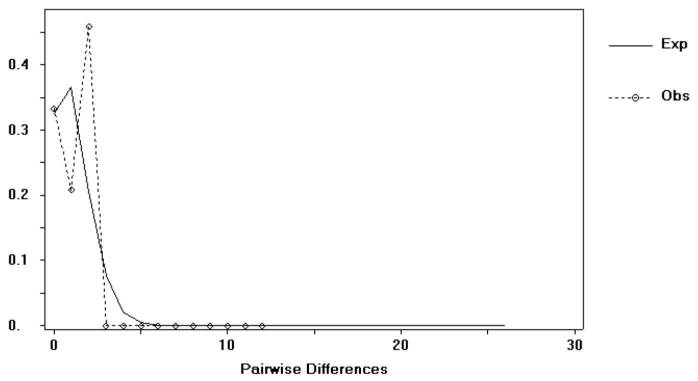

Table S1 Number of populations, locations, coordinates, chloroplast haplotypes and nuclear genotypes of *S. filiformis* and *S.* × *suecicus*.

Code	Locality	Longitude	Lattitude	Altitude	Haplotype (no. of individuals)	Genotype
1	River, Gongbujiangda, Xizang	E93°10'	N29°31'	3360m	Hap1 (7)	*S. filiformis*
2	Ditch, Duilongdeqing, Xizang	E90°58'	N29°39'	3678m	Hap2 (10)	*S. filiformis*
3	Pond, Mozugongka, Xizang	E91°39'	N29°48'	3740m	Hap1 (3), Hap3 (6)	*S. filiformis*
4	Marsh, Dazi, Xizang	E91°25'	N29°41'	3677m	Hap1 (8)	*S. filiformis*
5	River, Dangxiong, Xizang	E91°6'	N30°28'	4724m	Hap1 (4), Hap4 (4)	*S. filiformis*
6	Marsh, Deqin, Xizang	E90°56'	N30°23'	4176m	Hap1 (8)	*S. filiformis*
7	Pond, Nanmulin, Xizang	E89°18'	N29°23'	3948m	Hap1 (8)	*S. filiformis*
8	Canal, Mozugongka, Xizang	E91°52'	N29°46'	3895m	Hap1 (8)	*S. filiformis*
9	Pond, Linzhou, Xizang	E91°19'	N29°52'	3712m	Hap1 (4), Hap5 (4)	*S. filiformis*
10	River, Shiqunhe, Xizang	E80°5'	N32°29'	4264m	Hap6 (8)	*S. filiformis*
11	Pond, Kangma, Xizang	E89°38'	N28°48'	4089m	Hap1 (8)	*S. filiformis*
12	Pond, Rujiao, Saga, Xizang	E84°53'	N29°33'	4578m	Hap1 (8)	*S. filiformis*
13	Pond, Sangsang, Angren, Xizang	E86°40'	N29°26'	4557m	Hap1 (7)	*S. filiformis*
14	Pond, Saga, Xizang	E84°18'	N29°38'	4638m	Hap1 (8)	*S. filiformis*
15	River, Gongzhu, Xizang	E81°26'	N30°24'	4693m	Hap7 (8)	*S. filiformis*
16	River, Shaquqiao, Xizang	E85°14'	N29°25'	4679m	Hap7 (6)	*S. filiformis*
17	Marsh, Ritu, Xizang	E79°48'	N32°58'	4342m	Hap6 (9)	*S. filiformis*
18	Marsh, Qiongbuqiao, Xizang	E80°10'	N31°53'	4359m	Hap6 (8)	*S. filiformis*
19	Pond, Linzhi, Xizang	E94°32'	N29°34'	3354m	Hap1 (8)	*S. filiformis*
20	Pond, Gongjue, Xizang	E98°19'	N30°46'	3720m	Hap10 (8)	*S. filiformis*
21	Pond, Shade, Sichuan	E101°24'	N29°31'	3298m	Hap10 (8)	*S. filiformis*
22	River, Jiulong, Sichuan	E101°25'	N29°30'	3324m	Hap13 (8)	*S. filiformis*
23	Pond, Litang, Sichuan	E100°22'	N30°01'	4019m	Hap10 (8)	*S. filiformis*

24	Pond, Litang, Sichuan	E100°9'	N30°1'	3960m	Hap10 (8)	*S. filiformis*
25	River, Daocheng, Sichuan	E100°9'	N29°30'	3770m	Hap10 (10)	*S. filiformis*
26	Pond, Xiangcheng, Sichuan	E100°03'	N29°08'	4614m	Hap10 (8)	*S. filiformis*
27	Pond, Xinlong, Sichuan	E100°19'	N30°7'	4071m	Hap10 (4), Hap13 (4)	*S. filiformis*
28	Pond, Ganzi, Sichuan	E99°54'	N31°23'	4114m	Hap10 (6)	*S. filiformis*
29	Pond, Baiyu, Sichuan	E99°37'	N31°02'	3982m	Hap10 (8)	*S. filiformis*
30	Pond, Luhuo, Sichuan	E100°52'	N31°40'	3725m	Hap10 (8)	*S. filiformis*
31	Pond, Luhuo, Sichuan	E100°56'	N31°42'	3615m	Hap10 (8)	*S. filiformis*
32	Pond, Luhuo, Sichuan	E100°58'	N31°43'	3589m	Hap10 (6)	*S. filiformis*
33	Pond, Hongyuan, Sichuan	E102°33'	N32°05'	3414m	Hap13 (8)	*S. filiformis*
34	Pond, Hongyuan, Sichuan	E102°29'	N32°16'	3602m	Hap13 (6)	*S. filiformis*
35	Pond, Hongyuan, Sichuan	E102°29'	N32°17'	3617m	Hap13 (9)	*S. filiformis*
36	Pond, Hongyuan, Sichuan	E102°23'	N32°25'	3587m	Hap10 (8)	*S. filiformis*
37	Pond, Hongyuan, Sichuan	E102°19'	N32°35'	3529m	Hap10 (3), Hap13 (6)	*S. filiformis*
38	Pond, Hongyuan, Sichuan	E102°30'	N32°46'	3501m	Hap13 (8)	*S. filiformis*
39	Pond, Ruoergai, Sichuan	E102°18'	N33°10'	3461m	Hap10 (8)	*S. filiformis*
40	Pond, Ruoergai, Sichuan	E102°18'	N33°11'	3461m	Hap10 (8)	*S. filiformis*
41	Pond, Ruoergai, Sichuan	E102°32'	N33°20'	3464m	Hap10 (8)	*S. filiformis*
42	Pond, Huahu, Sichuan	E102°57'	N33°49'	3486m	Hap10 (6)	*S. filiformis*
43	Pond, Ruoergai, Sichuan	E102°36'	N33°09'	3466m	Hap10 (4), Hap13 (4)	*S. filiformis*
44	River, Haiyan, Qinghai	E101°2'	N36°48'	3444m	Hap8 (2), Hap9 (4)	*S. filiformis*
45	Daotang River, Qinghai	E100°44'	N36°34'	3069m	Hap10 (8)	*S. filiformis*
46	Pond, Jiangxigou, Qinghai	E100°16'	N36°37'	3180m	Hap8 (8)	*S. filiformis*
47	River, Xihai, Qinghai	E100°48'	N36°39'	3293m	Hap8 (8)	*S. filiformis*
48	Huangshui River, Huangyuan, Qinghai	E101°24'	N36°40'	2851m	Hap11 (8)	*S. filiformis*
49	Gahai Lake, Gannan, Gansu	E102°20'	N34°14'	3480m	Hap12 (5)	*S. filiformis*

50	Jinyu Lake, Geermu, Qinghai	E94°48'	N36°27'	2801m	Hap8 (5)	*S. filiformis*
51	Pond, Zhongdian, Yunnan	E99°48'	N27°48'	3410m	Hap10 (6)	*S. filiformis*
52	Daotang River, Qinghai	E100°45'	N36°34'	3143m	Hap10 (8)	*S. filiformis* × *S. pectinata* genotype AA
53	Marsh, Dongga, Xizang	E91°1'	N29°38'	3642m	Hap1 (8)	*S. filiformis* × *S. pectinata* genotype AA
54	Yuhe River, Lijiang, Yunnan	E100°13'	N26°53'	2435m	Hap10 (10)	*S. filiformis* × *S. pectinata* genotype BB

Table S2 Number of populations, locations, coordinates, chloroplast haplotypes and nuclear genotypes of *S. pectinata*.

Code	Locality	Longitude	Lattitude	Altitude	Haplotype (no. of individuals)	Genotype
55	Pond, Dashimo, Hulunbeier, Nei Monggol	E117°08'	N49°23'	661m	Hap14 (9)	*S. pectinata* genotype AA
56	Pond, Buerjin, Aletai, Xinjiang	E86°47'	N47°45'	462m	Hap15 (6)	*S. pectinata* genotype AA
57	Pond, Keluke, Delinha, Qinghai	E96°54'	N37°19'	2814m	Hap15 (6)	*S. pectinata* genotype AA
58	Pond, Jiangxigou, Qinghai	E100°16'	N36°37'	3180m	Hap16 (8)	*S. pectinata* genotype AA
59	Hulun Lake, Hulunbeier, Nei Monggol	E117°40'	N49°19'	540m	Hap18 (10)	*S. pectinata* genotype BB
60	Cibi Lake, Eryuan, Dali, Yunnan	E99°57'	N26°08'	2050m	Hap16 (8)	*S. pectinata* genotype BB
61	River, Manzhouli, Nei Monggol	E117°24'	N48°18'	558m	Hap16 (8)	*S. pectinata* genotype BB
62	Lashihai Lake, Lijiang, Yunnan	E100°08'	N26°52'	2440m	Hap16 (6)	*S. pectinata* genotype BB
63	Lugu Lake,Yanyuan, Sichuan	E100°54'	N27°43'	2692m	Hap16 (8)	*S. pectinata* genotype BB
64	Fuxian Lake, Yuxi, Yunnan	E102°50'	N24°24'	1721m	Hap17 (8)	*S. pectinata* genotype CC
65	Xiangsi Lake, Yanqi, Xinjiang	E86°36'	N42°02'	1052m	Hap15 (8)	*S. pectinata* genotype CC
66	Pond, Shawan, Tacheng, Xinjiang	E85°56'	N44°29'	385m	Hap19 (7)	*S. pectinata* genotype CC
67	River, Taixin, Chengdu, Sichuan	E104°12'	N30°45'	517m	Hap20 (8)	*S. pectinata* genotype CC
68	River, Huaimao, Jiuquan, Gansu	E98°34'	N39°50'	1776m	Hap21 (5)	*S. pectinata* genotype CC
69	Keluke Lake, Delinha, Qinghai	E96°53'	N37°19'	2815m	Hap19 (6)	*S. pectinata* genotype CC
70	Pond, Xining, Qinghai	E101°40'	N36°39'	2560m	Hap19 (5)	*S. pectinata* genotype CC
71	Pond, Jiangxigou, Qinghai	E100°15'	N36°36'	3180m	Hap16 (4), Hap22 (4)	*S. pectinata* genotype AC
72	Daotang River, Daotanghe, Qinghai	E100°45'	N36°34'	3140m	Hap16 (8)	*S. pectinata* genotype AC
73	Ditch,Wulunguhu, Aletai, Xinjiang	E87°44'	N47°17'	478m	Hap15 (6)	*S. pectinata* genotype AC
74	Dashimo, Hulunbeier, Nei Monggol	E117°06'	N49°21'	661m	Hap14 (6)	*S. pectinata* genotype AB
75	Changqiaohai Lake, Mengzi, Yunnan	E103°22'	N23°25'	1283m	Hap23 (8)	*S. pectinata* genotype BC
76	West Erhai Lake, Dali, Yunnan	E100°06'	N25°55'	1962m	Hap24 (8)	*S. pectinata* genotype BC
77	Pond, Qidian, Kunming, Yunnan	E102°57'	N24°56'	1938m	Hap15 (5)	*S. pectinata* genotype BC

78	River, Guiyang, Guizhou	E106°42'	N26°34'	1061m	Hap23 (6)	*S. pectinata* genotype BC
79	Jinhu Lake, Huaian, Jiangsu	E119°06'	N33°01'	13m	Hap23 (8)	*S. pectinata* genotype BC
80	Ditch, Shengshui, Hanzhong, Shanxi	E107°07'	N33°03'	543m	Hap23 (8)	*S. pectinata* genotype BC
81	Hongze Lake, Suqian, Jiangsu	E118°28'	N33°33'	16m	Hap15 (8)	*S. pectinata* genotype BC
82	Pond, Hanzhong, Shanxi	E107°12'	N33°30'	1173m	Hap23 (8)	*S. pectinata* genotype BC
83	Pond, Xiaoyizhuang, Dali, Yunnan	E100°11'	N25°42'	1968m	Hap24 (8)	*S. pectinata* genotype BC
84	East Erhai Lake, Dali, Yunnan	E100°12'	N25°41'	1962m	Hap15 (5), Hap25 (5)	*S. pectinata* genotype BC
85	Yangzhonghai Lake, Kunming, Yunnan	E102°59'	N24°55'	1768m	Hap16 (8)	*S. pectinata* genotype BC
86	Boluo River, Dali, Yunnan	E100°11'	N25°41'	1984m	Hap16 (8)	*S. pectinata* genotype BC
87	Changning River,Weinan,Shanxi	E109°51'	N35°22'	894m	Hap16 (6)	*S. pectinata* genotype BC

Table S3 Variable nucleotide sites in nuclear ITS sequences of *S. filiformis* and *S. pectinata*.

Taxon	0	0	0	0	0	0	0	1	1	1	1	1	1	1	1	2	3	4	4	4	5	5	5	5	6	6	6	6	6	6	6	6	6
	1	4	7	7	7	9	9	0	0	0	1	1	2	4	7	2	8	6	8	9	2	4	5	7	1	1	1	2	2	4	5	6	7
	5	8	1	4	5	5	6	3	4	9	1	3	7	1	4	6	8	3	8	5	6	9	8	4	1	2	4	6	7	1	0	7	0
S. filiformis	A	T	C	T	G	T	T	G	C	C	A	G	G	C	T	A	C	G	T	-	C	G	C	T	T	C	G	T	C	◎	C	T	G
S. pectinata genotype A	C	A	G	C	C	G	C	T	A	G	T	C	-	T	A	G	T	T	C	C	T	A	A	A	A	A	C	A	A	-	G	A	T
S. pectinata genotype B	C	A	G	C	G	G	C	G	C	G	T	C	-	T	A	G	C	T	C	C	C	G	A	A	T	C	G	A	A	-	G	A	T
S. pectinata genotype C	C	A	G	C	C	G	C	G	C	G	T	C	-	T	A	G	T	T	C	C	C	A	A	A	A	A	C	A	A	-	G	A	T

◎: ATTGTGGAT

Table S4 Variable nucleotide sites in 13 chloroplast haplotypes of *S. filiformis*.

Haplotype	Variable sites																												
	*atp*F-*atp*H			*rpl*20-*rps*12			*trn*D-*trn*T								*trn*S-*trn*G										*trn*L-*trn*F				
	3	3	4	1	5	5	0	0	0	0	1	1	4	4	0	0	1	1	2	2	2	2	3	4	0	0	3	4	6
	0	5	5	0	1	4	0	0	3	6	0	2	1	2	0	4	9	9	0	2	3	7	8	2	7	8	7	0	6
	1	2	6	9	0	8	5	6	2	6	8	2	8	8	9	4	6	7	7	1	9	6	6	7	5	2	2	2	3
Hap1	-	C	C	C	G	-	T	A	C	G	C	A	C	-	G	A	T	-	A	A	G	-	§	T	T	C	C	A	C
Hap2	-	C	C	C	T	-	T	A	C	G	C	A	A	-	G	A	T	※	A	A	G	-	§	T	T	C	C	G	C
Hap3	-	C	C	C	T	*	T	C	C	A	C	A	A	-	G	A	T	※	A	A	G	#	§	T	T	C	C	G	C
Hap4	-	C	C	C	G	-	T	A	C	G	C	A	C	-	G	A	T	-	A	T	G	-	§	T	T	C	C	A	C
Hap5	-	C	C	C	G	-	T	A	C	G	C	A	C	-	G	A	T	-	A	A	G	-	§	T	T	C	T	A	C
Hap6	-	C	C	C	T	*	T	C	C	G	C	A	A	-	G	A	T	※	A	A	G	-	§	T	T	T	C	G	C
Hap7	-	C	C	C	G	-	T	A	T	G	C	A	C	-	G	A	T	-	A	A	G	-	§	T	T	C	C	A	C
Hap8	A	T	C	T	T	*	C	A	C	G	T	A	C	◎	A	C	T	※	G	A	A	-	-	G	G	C	C	G	T
Hap9	A	T	C	T	T	*	C	A	C	G	T	A	C	◎	A	A	T	※	G	A	A	-	-	G	G	C	C	G	T
Hap10	A	T	C	T	T	*	C	A	C	G	T	A	C	◎	A	A	G	※	G	A	A	-	-	G	T	C	C	G	T
Hap11	A	T	T	T	T	*	C	A	C	G	T	A	C	◎	A	C	T	※	G	A	A	-	-	G	G	C	C	G	T
Hap12	A	T	T	T	T	*	C	A	C	G	T	A	C	◎	A	A	G	※	G	A	A	-	-	G	T	C	C	G	T
Hap13	A	T	C	T	T	*	C	A	C	G	T	C	C	◎	A	A	G	※	G	A	A	-	-	G	T	C	C	G	T

＃: TTTCTTCATTTACTTTCTGATT;

§: AATAAAAATAT;

※: CATGATTG;

◎: ATAATTG;

*: GATGAAAT

Table S5 Variable nucleotide sites in 12 chloroplast haplotypes of *S. pectinata*.

Haplotype	Variable sites										
	*trn*L-*rpl*32						*trn*Q-*rpS*16	*ndh*Ax1-*ndh*Ax2	*trn*C-*rpo*B		*psb*M-*trn*D
	241	327-330	331	471	515	536	307	375	491	693-726	519
Hap14	A	CTAT	A	C	T	A	C	C	A	-	T
Hap15	C	CTAT	A	C	T	A	C	A	A	-	C
Hap16	A	CTAT	A	C	T	A	C	A	A	-	T
Hap17	C	-	C	C	T	A	C	A	A	-	C
Hap18	A	CTAT	A	C	G	A	C	A	A	-	T
Hap19	C	CTAT	A	C	T	A	C	A	G	-	C
Hap20	A	-	A	C	T	A	T	A	A	-	C
Hap21	C	CTAT	A	T	T	A	C	A	G	-	C
Hap22	A	CTAT	A	C	T	C	C	A	A	#	T
Hap23	C	-	A	C	T	A	C	A	A	-	C
Hap24	A	-	A	C	T	A	C	A	A	-	C
Hap25	A	CTAT	A	C	T	A	C	A	A	-	C

#: CAATGAAAAATTAAATTCAATGAAAAATTAAATT

Table S6 Analysis of molecular variance (AMOVA) based on the cpDNA haplotypes of *S. filiformis* and *S. pectinata*.

Source of variation	Degrees of freedom	Sum of squares	Variance component	% Total variance	Fixation Index FST
**All populations of *S. filiformis***					0.964∗
Among populations	47	2211.119	11.360	96.37	
Within populations	149	63.733	0.428	3.63	
**Divided into two groups**					0.981∗
Among groups	1	1813.892	19.535	88.94	
Among populations within groups	46	397.227	2.001	9.11	
Within populations	149	63.733	0.428	1.95	
**All populations of *S. pectinata***					0.755∗
Among populations	30	287.027	1.907	75.54	
Within populations	115	71	0.617	24.46	
**Divided into three regions**					0.761∗
Among regions	2	38.236	0.197	7.60	
Among populations within regions	28	248.791	1.773	68.54	
Within populations	115	71	0.617	23.86	

∗P < 0.001, 1000 permutations.

Table S7 Results of the neutrality tests and the 95% confidence intervals of growth rates.

Lineage	Tajama's *D* (*P* value)	Fu's *Fs* (*P* value)	Growth rate
**All populations of *S. filiformis***	2.028 ($P > 0.05$)	6.517 ($P < 0.05$)	-485~872
The south group of *S. filiformis*	0.535 ($P >0.1$)	1.275 ($P >0.1$)	-464~971
The east group of *S. filiformis*	0.829 ($P >0.1$)	0.670 ($P >0.1$)	-472~970
**All populations of *S. pectinata***	0.245 ($P >0.1$)	-0.579 ($P >0.1$)	-473~985
The northwest region of *S. pectinata*	1.224 ($P >0.1$)	1.508 ($P >0.1$)	-469~979
The north region of *S. pectinata*	1.936 ($P >0.05$)	2.198 ($P >0.1$)	-472~973
The southwest region of *S. pectinata*	1.423 ($P >0.1$)	1.248 ($P >0.1$)	-470~976
